# Performance Evaluation, Optical Optimization and Earth-Based Validation of Star Sensors for Ground Detection in Martian Dust Environments

**DOI:** 10.3390/s26154686

**Published:** 2026-07-23

**Authors:** Yuan Gao, Ming-Jian He, Yan Li, Hong-Yuan Wang, Shun-Li Li, Hong Qi

**Affiliations:** 1 School of Astronautics, Harbin Institute of Technology, Harbin 150001, China; gaoyuanhtby@163.com (Y.G.); fountainhy@hit.edu.cn (H.-Y.W.); lishunli@hit.edu.cn (S.-L.L.); 2 Shanghai Aerospace Control Technology Institute, Shanghai 201109, China; 3 National Key Laboratory of Space Target Awareness, Shanghai 201109, China; 4 Shanghai Key Laboratory of Aerospace Intelligent Control Technology, Shanghai 201109, China; 5 School of Energy Science and Engineering, Harbin Institute of Technology, Harbin 150001, China; ly135285@163.com (Y.L.); qihong@hit.edu.cn (H.Q.); 6 Key Laboratory of Aerospace Thermophysics, Ministry of Industry and Information Technology, Harbin 150001, China

**Keywords:** radiative transfer, Martian dust, star sensor, performance evaluation, parameter optimization, ground detection

## Abstract

**Highlights:**

**What are the main findings?**

- Established a spectral atmospheric radiative transfer model for Martian dust based on the Null Collision Monte Carlo Method, validated against actual Mars rover Navcam observations with a 7.83% average relative error.
- Optimized key optical configurations (22 mm aperture, 18° FOV, and 100 ms integration time) and proposed a verified Earth-Mars radiative equivalence experimental scheme for robust performance evaluation.

**What are the implications of the main findings?**

- These findings confirm the feasibility of using star sensors for all-day, high-precision autonomous attitude determination on the Martian surface, addressing the limitations of IMU drift and sun sensor time constraints.
- This work provides a critical technical framework and parameter design reference for future deep-space missions requiring precise surface-based navigation, such as Mars sample return.

**Abstract:**

In deep-space exploration and remote sensing, characterizing radiative transfer in complex planetary atmospheres is fundamental for robust target detection and optical navigation. On the Martian surface, intense scattering and attenuation by dust aerosols pose severe environmental interference, challenging star sensors used for high-precision navigation. To address this, this study develops a spectral radiative transfer model based on the Null Collision Monte Carlo Method to characterize the optical background of the dusty Martian atmosphere. Mie scattering theory is employed for dust particles, while gas molecular absorption is modeled via line-by-line integration. The simulated sky radiance is validated against Mars rover Navcam observations, yielding an average relative error of 7.83% between the modeled and observed radiance values across scattering angles greater than 5°. Building on this, an imaging link model evaluates surface-based detection performance, including signal-to-noise ratio, detection success probability, and star count. Optical parameters—aperture, field of view, and integration time—are optimized for nighttime and dawn-dusk modes. Spatio-temporal assessments are conducted globally across Martian years, focusing on the Zhurong landing site and Tianwen-3 candidates. Finally, an Earth-environment equivalence experiment using a 60% transmittance filter verifies the design’s robustness. This work confirms the feasibility of star-sensor-based attitude determination on Mars.

## 1. Introduction

A star sensor is a high-precision navigation instrument capable of determining attitude by detecting starlight directions [1,2,3,4]. Due to its high accuracy, high update rate, and reliability, it has been widely utilized across sea, airborne, and space platforms [5,6,7,8,9,10,11,12,13]. Existing research primarily covers star map simulation, star point extraction, navigation catalog construction, star identification, and error calibration [3,14,15,16,17,18,19,20,21,22,23,24]. As the second country to successfully land a rover and conduct scientific missions on Mars, China has announced plans for a Mars sample return mission around 2030 [25], demanding unprecedented accuracy in initial attitude determination during the ascent phase. Although star sensors are crucial for orbital maneuvers, their deployment on the Martian surface remains unreported, leaving their surface detection feasibility unverified due to the significant environmental differences between Earth and Mars.

Mars surface exploration missions impose increasingly stringent requirements on attitude determination. On one hand, the Martian surface environment is exceptionally complex and dynamic, characterized by drastic diurnal temperature fluctuations, persistent dust activity, and severe deep-space command transmission latencies, requiring landers and rovers to possess high autonomy for stable and accurate attitude estimation [26,27]. Meanwhile, these surface probes face strict mass, volume, and power limitations, urgently requiring a lightweight, low-power, and high-precision orientation system.

Driven by these requirements, star sensors serve as an ideal choice due to their superior precision and autonomy. Currently, Martian rovers mainly rely on daylight-restricted sun sensors and drift-prone Inertial Measurement Units (IMUs) [28]. In contrast, star sensors provide all-day, high-precision absolute orientation independent of local magnetic fields or orbital links, yielding measurement accuracies several orders of magnitude higher than sun sensors. Providing absolute attitude with non-cumulative errors, they are vital in featureless terrains where visual navigation fails. Integrating star sensors with IMUs effectively suppresses dead-reckoning drift, ensuring high-fidelity attitude data for long-term surface operations and critical ascent maneuvers [26,27], which is essential for future sophisticated Mars missions such as sample return [29].

Unlike space-based platforms, surface-based star sensors experience performance degradation from starlight attenuation caused by atmospheric molecular absorption and aerosol scattering [30], a phenomenon well-documented on Earth [31,32,33,34,35]. Compared to Earth, the Martian atmosphere features lower gas molecular absorption due to a rarefied pressure (~1% of Earth’s) [36], but suffers severe transmittance attenuation from micron-scale suspended dust particles that increase the optical thickness [37]. The temperature profiles used in this study are based on the Mars Climate Database (MCD) under non-dusty conditions. Major dust storms are excluded from the current scope, as they simultaneously introduce large temperature perturbations and severe extinction effects that would require a dedicated analysis beyond the present work. The classic Mie scattering theory for spherical dust particles, combined with line-by-line integration for gas absorption (predominantly CO_2_), forms the foundational framework for Martian radiative transfer modeling [38,39]. Based on this framework and data from spectrometers onboard historical missions (such as IRIS on Mariner 9 and TES on Mars Global Surveyor), systematic simulations from visible to thermal infrared bands have retrieved key parameters such as temperature profiles, dust optical depths, single scattering albedos, and asymmetry factors [38,40], benchmarking dust optical properties with foundational models [38].

Furthermore, spatial and seasonal variations in Martian dust loading heavily regulate atmospheric transmittance. Utilizing multi-year solar imaging data from Mars Exploration Rovers (MER), studies have retrieved dust optical depths to reveal how regional and global dust storms modulate optical thickness and drive seasonal dust particle size evolution, with effective radii shifting from ~1 µm in quiet seasons to ~2 µm during storm events [40]. Extended records from Gale Crater based on Curiosity solar imaging confirm that optical depth peaks during southern spring and summer while remaining lower and stable in autumn and winter, noting that morning peaks correlate with thermal tides [41].

Although celestial-aided inertial navigation schemes (SINS/CNS) have been explored to correct gyro drifts and alignment errors via star tracking [42,43], these algorithm-level investigations lack systematic modeling and quantitative evaluation regarding how the dusty Martian radiative transfer process physically degrades starlight detection at the entrance pupil.

In summary, despite the unique advantages of star sensors as a promising surface solution, their capability under Martian surface conditions has not yet been systematically evaluated. Therefore, this study seeks to verify and assess star sensor performance on Mars through numerical simulations and ground-based equivalent experiments. Given that sensor quantum efficiency is highly spectrally sensitive [44], integrating spectrally varying atmospheric transmittance into the detection chain model is essential for meaningful signal-to-noise ratio (SNR) evaluations [45]. Bailey et al. showed that using a constant band-averaged transmittance (the traditional “standard star” method) introduces errors of a few percent for a solar-type star. This underscores that a spectral approach is essential for accurate signal evaluation [46]. In this study, numerical simulations are employed to calculate the spectral transmittance of the Martian atmosphere under dust conditions. This transmittance is then integrated into the detection chain model to evaluate the performance of star sensors on the Martian surface. Performance metrics include detection success probability, the number of detectable stars, and SNR. The study further optimizes the optical parameters of the star sensor—such as lens aperture, FOV, and integration time—under two operational modes: nighttime detection, dawn and dusk detection. A comprehensive performance assessment is conducted across Martian surface locations, considering various Martian years and seasons, from both spatial and temporal perspectives. This evaluation aims to demonstrate the feasibility of using star sensors for attitude determination on Mars, thereby providing optimized technical solutions for future Mars exploration missions.

## 2. Detection Chain Model for Mars Surface Dust Environment

### 2.1. Detection Chain Model of the Star Sensor

Stellar radiation can be approximated as blackbody radiation, with its spectral radiance expressed as:

(1) $$M(\lambda ,T)=\frac{c_{1}}{\lambda^{5}\left[\exp\left(c_{2}/\lambda T\right)-1\right]}$$

where λ is the wavelength, *c*_1_ is the first blackbody radiation constant, *c*_2_ is the second blackbody radiation constant, and *T* is the stellar temperature.

The blackbody radiation law describes the relative spectral distribution of stellar radiation. To obtain the absolute spectral distribution, a calibration is required. This involves multiplying the relative spectral distribution by a scaling factor to yield the absolute spectral distribution. For a star with a magnitude m and an effective temperature *T*, the absolute spectral distribution can be expressed as [47]:

(2) $$E_{m}(\lambda ,T)=\frac{E_{0}\cdot {\left(2.512\right)}^{-m}\cdot M(\lambda ,T)}{\int_{0.4}^{0.8}M(\lambda ,T)\cdot P_{eye}(\lambda )\cdot d\lambda }$$

The irradiance of a zero-magnitude star *E*_0_ is typically defined as *E*_0_ = 2.65 × 10^−6^ Lux, *P_eye_*(*λ*) is the photopic efficacy function, whose response range is 0.4–0.8 µm.

Based on the relationship between stellar irradiance and magnitude, the spectral radiant flux of a star with magnitude m, as received on the focal plane of the image sensor after passing through the optical system, is given by:

(3) $$\Phi_{m}(\lambda ,T)=\tau_{o}(\lambda )\cdot E_{m}(\lambda ,T)\cdot \pi \cdot {\left(\frac{D}{2}\right)}^{2}$$

where $\tau_{o}\left(\lambda \right)$ is the transmittance of the optical system (assumed to be 0.7 for calculation), $E_{m}(\lambda ,T)$ is the spectral irradiance of the stellar target, *D* is the effective aperture of the star sensor’s optical system.

As indicated by the aforementioned calculation formula for stellar photoelectrons [48], The stellar spectral flux depends on both apparent magnitude and color temperature, both of which are essential inputs to the SNR analysis.

The number of stellar target photons *N_ph_* received by the image sensor on the focal plane through the optical system during the integration time t is given by:

(4) $$N_{\mathrm{ph}}=\Phi_{m}(\lambda ,T)\cdot t\cdot \frac{1}{E_{\mathrm{ph}}}$$

where *E_ph_* is the energy of a single photon, given by *E_ph_* = *hc*/*λ*, *h* is Planck’s constant (*h* = 6.626 × 10^−34^ J·s), *c* is the speed of light (*c* = 3.0 × 10^8^ m/s), *t* is the integration time.

Considering the atmospheric transmission characteristics and the photoelectric conversion efficiency of the image sensor, the output signal charge *S* of the star sensor’s image sensor can be expressed as:

(5) $$S=\int_{\lambda_{1}}^{\lambda_{2}}N_{ph}\cdot \tau_{a}(\lambda )\cdot \eta (\lambda )d\lambda$$

where $\tau_{a}\left(\lambda \right)$ is the atmospheric transmittance, $\eta \left(\lambda \right)$ is the quantum efficiency of the image sensor.

This equation indicates that the stellar target signal energy received and converted by the star sensor is primarily influenced by the optical system’s aperture, the transmittance of the optical system, the integration time, the atmospheric transmittance, and the sensor’s quantum efficiency.

Based on the imaging link model described above, the background signal transfer and conversion can be modeled as [49]:

(6) $$N_{B}=\int_{\lambda_{1}}^{\lambda_{2}}B_{\lambda }\cdot \eta (\lambda )\cdot t\cdot \pi \cdot {\left(\frac{D}{2}\right)}^{2}\cdot {\left(\frac{a_{\mathrm{pixel}}}{f}\right)}^{2}d\lambda$$

where $B_{\lambda }$ is the number of background photons per unit spectral bandwidth, *a_pixel_* is the pixel size of the star sensor, f is the focal length of the optical system.

To ensure the reproducibility of the simulation results and the consistency of the imaging link model, the specific parameters of the reference star sensor used in this study are summarized here. The system employs a CMOS image sensor with a 2048 × 2048 pixel array and a pixel size of 6.5 μm. The readout noise is 28 e^−^ and the dark current density is 106 e^−^/s/pix. For the optical configuration, two effective apertures are considered: *D* = 22 mm and *D* = 30 mm, with a focal length f (as used in Equation (6)) = 42 mm, corresponding to the 18° field of view (FOV) verified in the subsequent experimental section. Furthermore, instead of adopting a simplified constant value, the quantum efficiency *η(λ)* utilized in Equation (5) follows the actual spectral response curve of the CMV4000 sensor (detailed in Section 5), covering a detection range of 400–1000 nm. These specifications are consistently maintained across both the theoretical SNR evaluations and the Earth-equivalent validation experiments.

### 2.2. Spectral Transmittance of Mars’ Atmosphere in Dusty Conditions

Starlight traveling from the infinite reaches of space to the surface of Mars is attenuated by the presence of gas molecules (e.g., carbon dioxide) and dust particles in the Martian atmosphere. This attenuation can be divided into two components: absorption and scattering. The atmospheric transmittance *τ*(*λ*) can be mathematically expressed as:

(7) $$\tau \left(\lambda \right)=\exp\left(-\left(\alpha \left(\lambda \right)+\beta \left(\lambda \right)\right)L\right)$$

where $\alpha \left(\lambda \right)$ is the spectral absorption coefficient, $\beta \left(\lambda \right)$ is the spectral scattering coefficient, L is the path length of light propagation through the atmosphere.

In this study, Martian dust particles are assumed to be spherical, and their interactions with starlight are characterized by Mie scattering theory. While actual dust particles are often irregular, preliminary T-matrix simulations for Chebyshev-type particles—which represent the non-spherical geometries closest to spheres—showed that deviations in extinction cross-sections and asymmetry factors from the Mie-based spherical model are not significant for the spectral range of interest [50]. Given this and the broad adoption of Mie theory in Martian atmospheric studies [51,52], the spherical approximation is employed. The minor fluctuations in the spectral results arise from interference effects in the size distribution integration and do not affect the SNR predictions. Notably, molecular scattering (Rayleigh scattering) is treated as the wavelength limit of the spherical particle scattering formulation.

The volume scattering coefficient under standard conditions is given by:

(8) $$k_{sc,0,\lambda }=1.0563\times 10^{-6}\lambda^{-4}$$

where the wavelength unit is μm, the unit of $k_{sc,0,\lambda }$ is m^−1^.

The interaction of gas molecules with starlight involves Rayleigh scattering and spectrally selective absorption. The spectrally selective absorption of gas molecules is modeled using the Planck-weighted narrow band approach [53]. In this study, the Planck function weighting within a narrow spectral band is considered. Within a narrow spectral interval (w−Δw/2,w+Δw/2) around any center wavenumber w, the Planck-weighted spectral absorption coefficient can be expressed as:

(9) $$\kappa_{P,\;k}=\frac{\int_{w_{1}}^{w_{2}}\kappa_{w}I_{bw}\;dw}{\int_{w_{1}}^{w_{2}}I_{bw}\;dw}$$

where *k* represents the *k*-th wavenumber interval, $w_{1}∼w_{2}$ denotes the corresponding narrow spectral band, $\kappa_{w}$ is the high-resolution spectral absorption coefficient of the gas; and $I_{bw}$ is the spectral blackbody radiative intensity determined by the local atmospheric temperature.

Since the narrow band absorption coefficient is the sum of contributions from individual spectral lines, Equation (9) can be rewritten as:

(10) $$\kappa_{P,\;k}=\frac{\sum_{i}\int_{w_{1}}^{w_{2}}\kappa_{iw}I_{bw}\;dw}{\int_{w_{1}}^{w_{2}}I_{bw}\;dw}=\frac{\sum_{i}I_{bw_{i,\;k}}S_{i,\;k}}{\int_{w_{1}}^{w_{2}}I_{bw}dw}$$

where *i* represents the index of an individual spectral line within the *k*-th narrow band; $\kappa_{iw}$ is the high-resolution spectral absorption coefficient contributed solely by the *i*-th single line; $S_{i,k}$ is the integrated line strength of the *i*-th spectral line; and $I_{bw_{i,k}}$ denotes the specific blackbody radiative intensity evaluated at the center wavenumber of the *i*-th spectral line within the *k*-th narrow band.

The Planck-weighted narrow-band radiative property database for gas molecules is developed using the latest HITRAN2020 dataset. High-resolution spectral absorption coefficients are first obtained using the line-by-line integration method, and the Planck-weighted narrow-band absorption coefficients are then derived accordingly [54,55].

The spectral extinction, scattering, and absorption factors of particles are described by Lorenz-Mie electromagnetic theory as follows:

(11) Qext, λ=2x2Re∑n=1∞2n+1an+bn

(12) $$Q_{\mathrm{sca},\;\lambda }=\frac{2}{x^{2}}\sum_{n=1}^{\infty }\left(2n+1\right)\left({\left|a_{n}\right|}^{2}+{\left|b_{n}\right|}^{2}\right)$$

(13) $$Q_{\mathrm{abs},\;\lambda }=Q_{\mathrm{ext},\;\lambda }-Q_{\mathrm{sca},\;\lambda }$$

where Re denotes the real part of a complex number, *x* = *πD*/*λ* is the dimensionless size parameter, *D* is the particle diameter, *λ* is the wavelength, *a_n_* and *b_n_* are the Mie scattering coefficients, expressed as:

(14) $$\begin{array}{l} a_{n}=\frac{\psi_{n}^{\prime }(mx)\psi_{n}(x)-m\psi_{n}(mx)\psi_{n}^{\prime }(x)}{\psi_{n}^{\prime }(mx)\xi_{n}(x)-m\psi_{n}(mx)\xi_{n}^{\prime }(x)}\\ b_{n}=\frac{m\psi_{n}^{\prime }(mx)\psi_{n}(x)-\psi_{n}(mx)\psi_{n}^{\prime }(x)}{m\psi_{n}^{\prime }(mx)\xi_{n}(x)-\psi_{n}(mx)\xi_{n}^{\prime }(x)} \end{array}$$

where $\psi_{n}(x)$ and $\xi_{n}(x)$ represent the Riccati-Bessel functions of the first and second kind, respectively, with the primes denoting their first derivatives; and $m=m_{r}-i^{\prime }m_{i}$ is the complex refractive index of the dust particle relative to the surrounding medium, where $m_{r}$ and $m_{i}$ represent the real and imaginary parts, respectively, with *i*′ denoting the imaginary unit.

The absorption, scattering, and extinction cross-sections of an individual particle (denoted as $C_{\mathrm{abs}}$, $C_{\mathrm{sca}}$, and $C_{\mathrm{ext}}$, respectively) are determined by scaling their corresponding efficiencies with the particle’s geometric cross-sectional area:

(15) $$C_{\mathrm{abs}}(D)=Q_{\mathrm{abs},\lambda }\cdot \frac{\pi D^{2}}{4}$$

(16) $$C_{\mathrm{sca}}(D)=Q_{\mathrm{sca},\lambda }\cdot \frac{\pi D^{2}}{4}$$

(17) $$C_{\mathrm{ext}}(D)=Q_{\mathrm{ext},\lambda }\cdot \frac{\pi D^{2}}{4}$$

If the particle number density *N*(*D*) is known, the macroscopic absorption coefficient $\kappa_{\mathrm{dust}}$, scattering coefficient $\sigma_{\mathrm{dust}}$, and extinction coefficient $\beta_{\mathrm{dust}}$ for the total dust particle ensemble can be determined by integrating across the entire size range:

(18) $$\kappa_{\mathrm{dust}}=\int_{0}^{\infty }C_{\mathrm{abs}}(D)N(D)dD$$

(19) $$\sigma_{\mathrm{dust}}=\int_{0}^{\infty }C_{sca}\left(D\right)N\left(D\right)dD$$

(20) $$\beta_{\mathrm{dust}}=\int_{0}^{\infty }C_{ext}\left(D\right)N\left(D\right)dD$$

As indicated above, calculating the absorption of gas molecules requires knowledge of the temperature, pressure, and concentration profiles of different gas components within the atmosphere as functions of altitude [56]. The complex refractive index of the dust particles is taken from literature [57].

**Figure 1:**
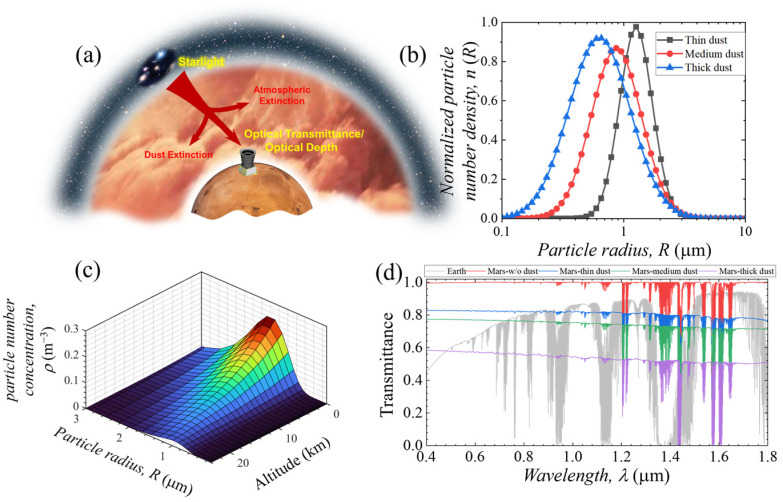
Martian atmospheric radiative transfer model and spectral transmittance characteristics. (**a**) Schematic illustration of starlight transmission through Martian atmospheric dust and nighttime detection by a star sensor on Mars surface. (**b**) Particle size distribution at 0 km altitude above the Martian surface under thin, medium, and thick dust conditions. (**c**) Particle number concentration distribution as a function of distance from the Martian surface and particle radius. (**d**) Atmospheric transmittance spectra of Earth and Mars under different dust conditions.

Figure 1a shows a schematic diagram of starlight propagation under the influence of Martian atmospheric dust and the nighttime detection of star sensors on the Martian surface. The starlight emitted by the star interacts with the gases and dust particles in the Martian atmosphere before reaching the entrance aperture of the star sensor. Both the Martian atmosphere and dust particles attenuate the starlight, which is reflected in the optical transmittance or optical depth of the transmission process. The terrain and morphology in different regions on Mars have varying atmospheric dynamics, resulting in differences in dust concentration, particle size, and distribution. In 1995, Chassefière et al. analyzed the Auguste occultation data from Phobos 2 and derived vertical profiles of particle number concentration and effective radius as a function of altitude, with the effective variance denoted as b [58]. According to this work, the particle number concentration and effective radius at different altitudes on Mars are categorized into three cases—thin dust, medium dust, and thick dust—whose representativeness in the Martian environment will be discussed in Section 4. As shown in Figure 1b, the particle size distribution functions of the three types of dust at 0 km above the Martian surface (which is considered equivalent to an altitude of 0 km in the simulation) are presented. A log-normal distribution function is employed to fit the particle size distribution of Martian dust particles, with the ordinate representing the normalized number density such that the integral over all radii equals unity. Figure 1c shows the particle number concentration distribution at different altitudes above the Martian surface under thin dust conditions, with varying particle radius. The subsequent simulation analysis uses the altitude profiles of particle size and concentration under the three dust conditions.

Figure 1d shows the spectral distribution of optical transmittance on the surface of Earth and Mars at sea level (0 km altitude), with the star sensor’s optical axis zenith angle set to 0°. The gray lines represent the results for Earth’s atmosphere, using the typical 1976 US Standard atmospheric model, with aerosols selected from the Rural-VIS = 23 km model. To quantitatively illustrate and compare the impact of Martian atmospheric molecules and dust on optical transmittance, the red curve specifically shows the optical transmittance on considering only the Rayleigh scattering and selective absorption effects of Martian atmospheric molecules (assuming no dust). This idealized scenario provides a reference for assessing the additional attenuation contributed by Martian dust loading.

From the results shown in Figure 1, it can be seen that without considering the impact of Martian dust, the atmospheric transmittance on Mars is relatively high, particularly in the visible light region, where selective absorption and Rayleigh scattering by atmospheric molecules are negligible, making the spectral transmittance close to 100%. In the shortwave infrared band, due to the selective absorption of atmospheric molecules, there are several distinct selective absorption peaks in the spectral transmittance. By incorporating the three different levels of dust parameters from Figure 1b into Equations (7–20) for simulation, three distinct optical transmittance distributions are generated, represented by blue, green, and purple curves in Figure 1d. From Figure 1, attenuation of starlight caused by Mie scattering of dust has a relatively weak correlation with wavelength, and the optical attenuation caused by Mie scattering increases slightly with increasing wavelength. Additionally, variations in dust particle size and concentration lead to significant changes in the optical transmittance of the entire Martian atmosphere. In the visible light region, the optical transmittance spans approximately 55% to 85%. This range essentially covers the optical attenuation caused by suspended dust in the Martian atmosphere under normal conditions, without the occurrence of Martian dust storms.

Regardless of the spectral variation dimension or the numerical scale, the optical transmittance of the Martian environment and Earth’s environment shows significant differences. Since the photoelectric detection chip in star sensors exhibits a noticeable spectral dependency in its photoelectric conversion efficiency (reflected in the quantum efficiency curve), this spectral dependency interacts with the optical transmittance of the detection link. Therefore, conducting detailed simulation calculations of the Martian atmospheric transmittance in the spectral dimension is essential for evaluating whether star sensors can successfully detect in the Martian dust environment.

**Figure 2:**
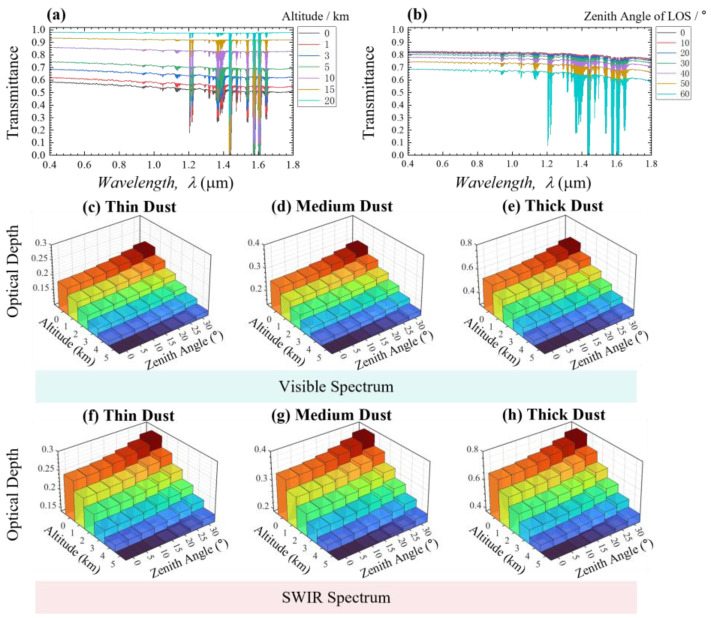
Atmospheric transmittance and optical depth on Mars under different altitudes, zenith angles, and dust loadings.

Figure 2a,b further quantitatively demonstrate the impact of the altitude of the star sensor and the zenith angle of the detection line of sight on the detection optical transmittance. Figure 2a shows the atmospheric transmittance spectral distribution at different altitudes on Mars under thick dust conditions (with the star sensor’s line-of-sight zenith angle at 0°). Figure 2b shows the variation in atmospheric transmittance spectrum distribution with different star sensor’s line-of-sight zenith angles at an altitude of 0 km above the Martian surface under thin dust conditions. For a given altitude and dust loading, the attenuation follows the Beer–Lambert–Bouguer law, where the slant optical depth increases with the zenith angle as $\tau (\theta )=\tau_{0}\cdot \sec(\theta )$ under the plane-parallel atmosphere assumption, and the corresponding transmittance is $T(\theta )=\exp[-\tau (\theta )]=\exp[-\tau_{0}\cdot \sec(\theta )]$. As a result, increasing the zenith angle lengthens the effective path through the dusty atmosphere, leading to the monotonic decrease in transmittance observed in Figure 2b, consistent with the trends reported for Martian dust extinction.

To further analyze the impact of star sensor on the attenuation of starlight in different optical spectral bands, Figure 2c–e and Figure 2f–h respectively present the calculated average optical depth values for the visible band (0.4–0.8 µm) and the short-wave infrared band (0.9–1.8 µm) under different altitudes and different detection line-of-sight zenith angles of the star sensor. The results shown in the figure indicate that, in both the visible and short-wave infrared bands, the optical depth monotonically decreases with increasing altitude and monotonically increases with increasing detection line-of-sight zenith angle. Under the same detection conditions, the optical depth in the short-wave infrared band is approximately 0.1 higher than that in the visible band. Therefore, it can be seen that selecting the visible band is a better choice under nighttime detection mode.

## 3. Radiative Transfer Model Validation and Optical Parameter Optimization for Martian Surface Star Sensors

### 3.1. Nighttime Detection Mode

Assuming the light spot is uniformly dispersed across *K* pixels (with *K* = 9 for calculation purposes), and denoting the signal photoelectron count per pixel when detecting a star of magnitude m as *S*/*K*, the simplified expression for the target SNR of the star sensor when detecting a star of magnitude *m* is given by:

(21) $$SNR=\frac{S\cdot \frac{1}{K}}{\sqrt{S\cdot \frac{1}{K}+N_{B}+N_{D}\cdot t+\sigma_{\mathrm{read}}^{2}}}$$

where *N_D_* is the dark current signal density, $\sigma_{\mathrm{read}}$ is the readout noise, and *N_B_* denotes the background photoelectron count per pixel [59].

Based on the results and analysis shown in Figure 2, it is evident that the altitude of the detector and the zenith angle of the detection line of sight, as well as the size and concentration of dust particles, all have a significant impact on the attenuation of starlight. Therefore, a more stringent observational condition was selected for the design of optical parameters and the analysis of SNR in the subsequent study. Unless otherwise specified, the altitude of the star sensor will be set at 0 km, the zenith angle of the detection line of sight at 30° (comprehensively considering mechanical rotation constraints and the horizon position), and the dust condition will be set as thick dust.

**Figure 3:**
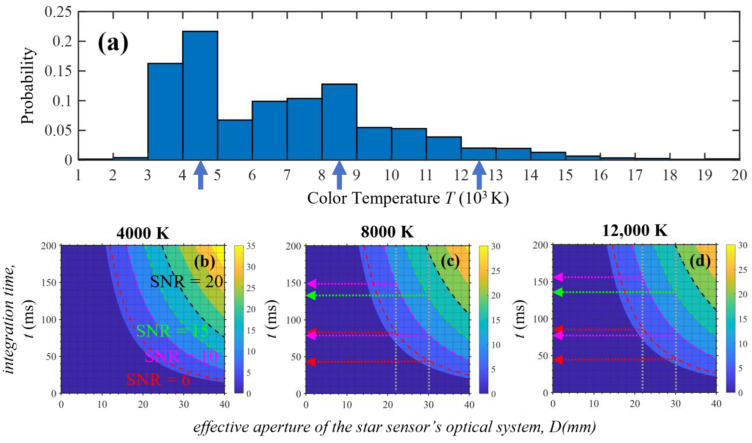
Selection of representative stellar color temperatures from the Hipparcos catalog and the corresponding SNR responses of 6th-magnitude stars to different aperture and integration time settings.

To select representative color temperature values, Figure 3a presents the probability distribution of color temperatures for stars with a magnitude ≥ 6 from the Hipparcos catalog. From the figure, it can be observed that the color temperature for stars in this magnitude range spans from 1000 K to 20,000 K. To ensure that the subsequent SNR analysis results are more representative and the optical parameter selections are better suited, three color temperatures—4000 K, 8000 K, and 12,000 K—will be analyzed.

To quantitatively analyze the impact of the integration time and the effective aperture size of the star sensor’s optical lens on detection performance, Figure 3b–d calculates the SNR for stars of magnitude 6 at 4000 K, 8000 K, and 12,000 K, respectively. Each figure displays contour lines for SNRs = 6, 10, 15, and 20. The results indicate that as integration time and effective aperture size increase, the detection SNR improves. However, in the actual design process of star sensor optical parameters, it is necessary to consider the mutual constraints and influences of multiple factors such as detection performance and the weight of the single unit. Therefore, it is not feasible to solely rely on increasing the integration time and effective aperture to enhance the detection performance of the star sensor. Subsequent analyses will consider two sets of parameters: effective aperture sizes of 22 mm with an integration time of 100 ms and effective aperture sizes of 30 mm with an integration time of 50 ms.

The two aperture sizes (22 mm and 30 mm) are selected as typical values balancing optical performance with mass constraints. Specifically, 22 mm represents a light and compact configuration that balances performance and weight [60], while 30 mm is a reasonable choice for moderately increasing aperture size within allowable limits to enhance detection sensitivity. For each aperture, the integration time is chosen to ensure SNR > 6 for a 6th-magnitude star under representative conditions (zenith angle 30°, thick dust), as verified from the contour plots in Figure 3. These two configurations serve as baseline cases for subsequent performance evaluation.

**Figure 4:**
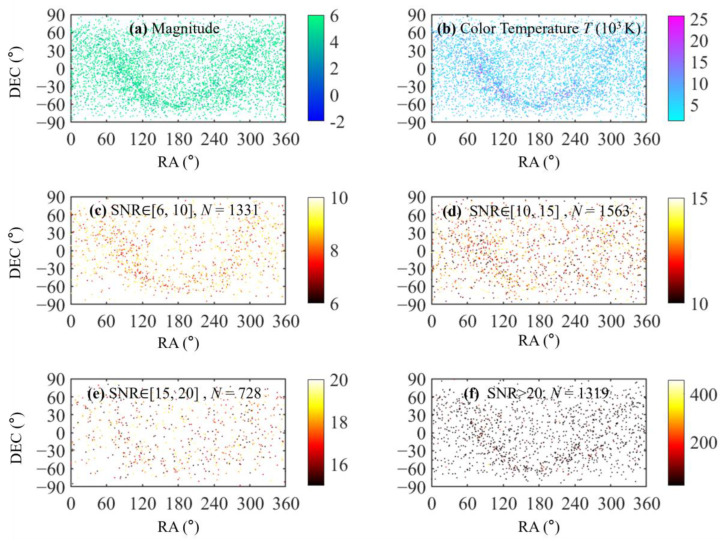
Celestial distribution of Hipparcos stars by magnitude, color temperature, and SNR under the baseline optical configuration (*D* = 22 mm, *t* = 100 ms).

To further substantiate the rationality of the aforementioned optical parameter design, the subsequent section conducts a SNR analysis for stars across the entire celestial sphere. Figure 4a,b illustrate the magnitude and color temperature distributions of stars from the Hipparcos catalog in celestial coordinates, serving as input parameters for a comprehensive star mapping survey. Using a star sensor with an effective aperture of 22 mm and an integration time of 100 ms, the SNR analysis of stars brighter than magnitude 6 from the catalog is shown in Figure 4c–f. There are 1331 stars with an SNR between 6 and 10, 1563 stars with an SNR between 10 and 15, 728 stars with an SNR between 15 and 20, and 1319 stars with an SNR greater than 20.

**Figure 5:**
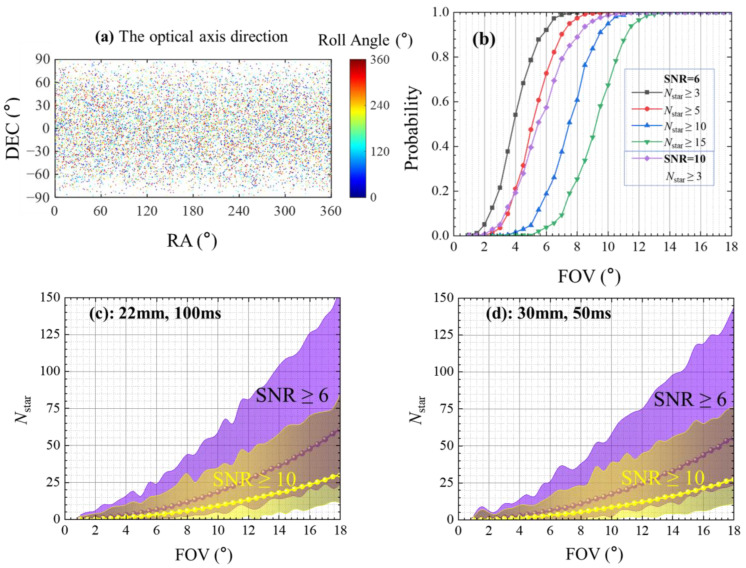
Statistical detection performance from 10,000 random pointings: FOV-dependent detection probability and detectable star counts for two optical configurations.

The aforementioned analysis considers only the SNR analysis method for magnitude 6 stars under a given color temperature, based on specified effective aperture and integration time conditions. However, to ensure the effective functionality of the star sensor, at least three observable stars must be present within the FOV. This necessitates the introduction of the FOV parameter as an additional dimension to enable a more comprehensive and rational optical parameter design. To address this, this paper conducts a simulation of 10,000 random star observations in the celestial coordinate system, with an effective aperture of 22 mm and an integration time of 100 ms. Figure 5a shows the right ascension, declination, and roll angle of the star sensor’s optical axis for these 10,000 observations. Figure 5b illustrates the probability of detecting at least *N_star_* stars within the FOV that can achieve the SNR threshold (SNR = 6 or SNR = 10) under different FOV conditions. The results show that as the FOV increases, the sensor’s lens covers a larger area of the sky, thus improving the likelihood of successful detection. At an SNR threshold of 6, a FOV of 8° can achieve 100% successful detection with at least 3 stars in the FOV. A FOV of 10° can achieve 100% success with at least 5 stars, a FOV of 12° can achieve 100% success with at least 10 stars, and a FOV of 14° can achieve 100% success with at least 15 stars. If the SNR threshold is set to 10, a FOV of at least 12° is required to achieve 100% success with at least 3 stars in the FOV.

Figure 5c,d provide an alternative perspective on the detection results from 10,000 random star observations, under different FOV conditions. These results are presented for two sets of optical design parameters: an effective aperture of 22 mm with an integration time of 100 ms, and an effective aperture of 30 mm with an integration time of 50 ms. The plots show the maximum and minimum number of stars detected within the FOV that satisfy the SNR ≥ 6 and SNR ≥ 10 conditions (represented by the transparent contour lines), as well as the average number of stars detected under these conditions in the 10,000 random observations (represented by the solid lines with filled dot). From the results, it can be seen that the SNR outcomes for the two design parameters do not differ significantly. A smaller aperture requires a longer integration time to achieve the same level of SNR. For low dynamic range detection scenarios on the Martian surface, a smaller aperture with a longer integration time can be selected. This approach helps minimize the instrument’s weight without compromising precision, which is highly meaningful for deep-space exploration missions.

The aforementioned results can actually provide a data foundation for the compilation of navigation star catalogs oriented towards Mars exploration. In fact, by calculating the total number of stars that meet the detection SNR and combining it with the given FOV of the star sensor, one can roughly estimate the expected number of stars within the FOV that satisfy the detection SNR threshold. However, even if the star sensor remains stationary on the Martian surface, the positions and quantities of stars within the field will change over time. To comprehensively assess the feasibility of star detection and determine the limits of optical parameter design, a full-sky survey approach is necessary for SNR analysis.

### 3.2. Dawn and Dusk Detection Mode

**Figure 6:**
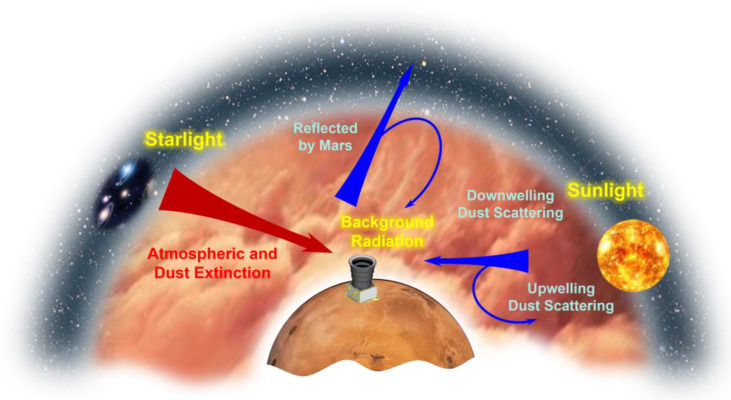
Schematic diagram of a star sensor on the Martian surface during dawn and dusk.

To further explore the performance limits of star sensors working on the Martian surface and to adapt to the complex Martian working environment, this paper presents a simulation test of detection performance and optical parameter optimization under dawn and dusk conditions (i.e., when the solar zenith angle is 90°). Figure 6 illustrates the schematic diagram of the star sensor’s detection during dawn and dusk conditions on the Martian surface. The transmission of starlight is still attenuated due to the absorption effect of gases and dust in the Martian atmosphere, characterized by the atmospheric transmittance. Unlike nighttime detection, under dawn and dusk conditions, although the Sun is at the horizon altitude, the unique nature of this critical scenario necessitates consideration of the radiance of the sky background caused by the interaction of solar radiation within the Martian atmosphere with atmospheric gas molecules and dust particles. This sky background radiance serves as background noise for the star sensor, significantly impacting its detection performance.

To solve the skyglow brightness caused by the interaction of Martian dust and gas molecules with sunlight, this paper employs Null Collision Monte Carlo Method (NCMCM) to simulate atmospheric radiation on Mars [61]. In traditional Monte Carlo methods used for radiative transfer simulations, the process first involves sampling the propagation distance *l* of light beams based on Beer-Lambert law. Then, the cumulative optical thickness is calculated along the path until the cumulative optical thickness equals the sampled distance *l*. Clearly, when the optical thickness distribution of the medium is non-uniform, the computational efficiency of traditional Monte Carlo methods decreases significantly. To balance calculation accuracy and efficiency while considering the unique altitude distribution of Martian dust particles, the Monte Carlo method is improved by introducing the developed NCMCM to simulate radiative transfer.

To evaluate the skyglow brightness caused by the interaction of Martian dust and gas molecules with sunlight under dawn and dusk conditions, the governing mathematical physics equations must be established. The baseline atmospheric background radiation is governed by the differential form of the monochromatic Radiative Transfer Equation (RTE), which describes the variation in spectral radiative intensity along a specific direction s, expressed as:

(22) $$s\cdot \nabla I_{\lambda }(r,s)=\kappa_{a,\lambda }I_{b,\lambda }(r)-(\kappa_{a,\lambda }+\kappa_{s,\lambda })I_{\lambda }(r,s)+\frac{\kappa_{s,\lambda }}{4\pi }\int_{4\pi }I_{\lambda }(r,s^{\prime })\Phi_{\lambda }(s^{\prime },s)d\Omega^{\prime }$$

where $I_{\lambda }(r,s)$ is the spectral radiative intensity at position r along direction s; $\kappa_{a,\lambda }$ and $\kappa_{s,\lambda }$ denote the total spectral absorption and scattering coefficients of the mixed atmosphere at wavelength λ, respectively; $\Phi_{\lambda }(s^{\prime },s)$ is the spectral scattering phase function; $\Omega^{\prime }$ is the solid angle; and $I_{b,\lambda }(r)$ represents the spectral blackbody radiative intensity determined by the local atmospheric temperature, which has been conceptually introduced earlier in Section 2.2.

Due to the highly non-uniform altitude distribution of Martian dust, traditional Monte Carlo tracking based on the differential RTE is computationally inefficient. To resolve this, the null-collision algorithm [61] is introduced by adding a fictitious coefficient $\kappa_{n,\lambda }$ to render the total extinction space-uniform, transforming the governing RTE into:

(23) $$\begin{array}{l} s\cdot \nabla I_{\lambda }(r,s)=\kappa_{a,\lambda }I_{b,\lambda }(r)-(\kappa_{a,\lambda }+\kappa_{s,\lambda }+\kappa_{n,\lambda })I_{\lambda }(r,s)\\ +\frac{\kappa_{s,\lambda }}{4\pi }\int_{4\pi }I_{\lambda }(r,s^{\prime })\Phi_{\lambda }(s^{\prime },s)d\Omega^{\prime }+\kappa_{n,\lambda }\int_{4\pi }I_{\lambda }(r,s^{\prime })\delta (s^{\prime },s)d\Omega^{\prime } \end{array}$$

where $\kappa_{n,\lambda }$ is the spectral null-collision coefficient, and $\delta (s^{\prime },s)$ is the Dirac delta function representing forward-scattering without physical deviation. Following the framework proposed by Galtier [62,63], Equation (23) can be formally rephrased as a Fredholm integral equation, which facilitates unbiased photon sampling in inhomogeneous media. Finally, the expectation of the backward-traced spectral radiative intensity is statistically determined as follows:

(24) $$I_{\lambda }(r,s)=\frac{1}{N}\sum_{j=1}^{N}I_{b,\lambda ,j}(r^{\prime })$$

where *N* is the total number of sampled energy bundles, and $I_{b,\lambda ,j}(r^{\prime })$ represents the specific spectral blackbody radiative intensity at the spatial position $r^{\prime }$ where the *j*-th energy bundle is absorbed or emitted.

When calculating skyglow brightness, the surface albedo of Mars is also required. The value is taken as the average between bright and dark regions, as cited from literature [64]. Additionally, the solar constant is another essential physical quantity used. Due to its seasonal variation on Mars, it ranges roughly between 400~700 W/m^2^. To ensure the generality of the analysis, this paper selects a value of 500 W/m^2^.

**Figure 7:**
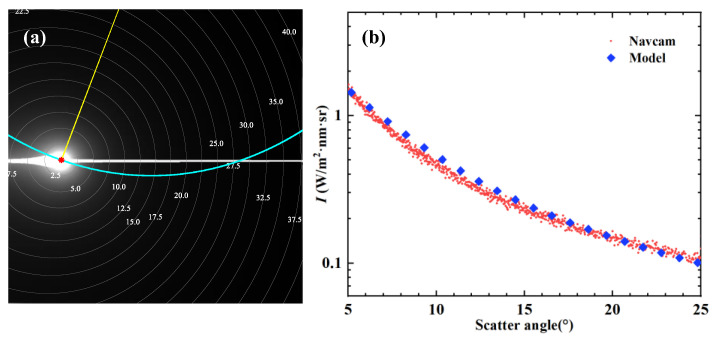
Radiance calibration results based on Mars rover Navcam images [65,66]. The yellow line is the line connecting the Sun and the zenith direction, and the blue line is the line connecting points with the same zenith angle as the Sun but different azimuth angles. The white concentric circles represent the contours of points with constant scattering angles.

To validate the accuracy of the proposed radiative transfer model and the star sensor’s performance under Martian conditions, the model-simulated radiance values are compared with those derived from actual Mars rover Navcam observations. It is important to note that the raw Navcam observations do not contain any dust aerosol parameters; the dust optical properties used in the NCMCM simulations are obtained from independent sources (e.g., the Mars Climate Database v6.1). Figure 7 presents the radiance calibration results derived from images acquired by the Mars rover Navcam. The CAHVOR [65,66] camera model is employed to perform geometric correction of the original images, establishing the mapping between pixel coordinates and viewing directions, and thereby determining the corresponding zenith angle, azimuth angle, and scattering angle for each pixel. Subsequently, the raw digital number (DN) values [67,68,69] are corrected for bias, dark current, and flat-field nonuniformity using the in-flight radiometric calibration coefficients. Combined with the exposure time, the DN values are converted into radiance with physical units of W·m^−2^·nm^−1^·sr^−1^.

During the radiance extraction process, as illustrated in Figure 7a, pixel samples are selected along a direction of monotonically increasing zenith angle at a fixed azimuth angle, using the solar direction as a reference. In Figure 7b, the red dots represent radiance values retrieved from Navcam observations, while the blue solid diamonds denote the corresponding model-simulated results. The two datasets exhibit good agreement in their overall trends, with radiance decreasing monotonically with increasing zenith angle, consistent with the path-length enhancement effect predicted by atmospheric radiative transfer theory. Since scattering angles smaller than 5° are significantly affected by the solar aureole, only data with scattering angles greater than 5° are considered in the subsequent error analysis, yielding an average relative error of 7.83% (calculated as the mean of the errors across different scattering angles, where each scattering angle error is averaged over multiple pixels), with the maximum deviation of 29.3% occurring at a scattering angle of 9°. This relative error is defined as the absolute difference between the modeled and observed radiance values, divided by the observed radiance.

Regarding the spectral range, the Navcam central wavelength is 675 nm, which lies within the operating spectral range of our star sensor (0.4–1.1 µm). This validation does not directly validate the star sensor itself, but rather the underlying radiative transfer model. Direct verification under simulated Martian dust conditions is provided by ground-based equivalent experiments (Section 5).

**Figure 8:**
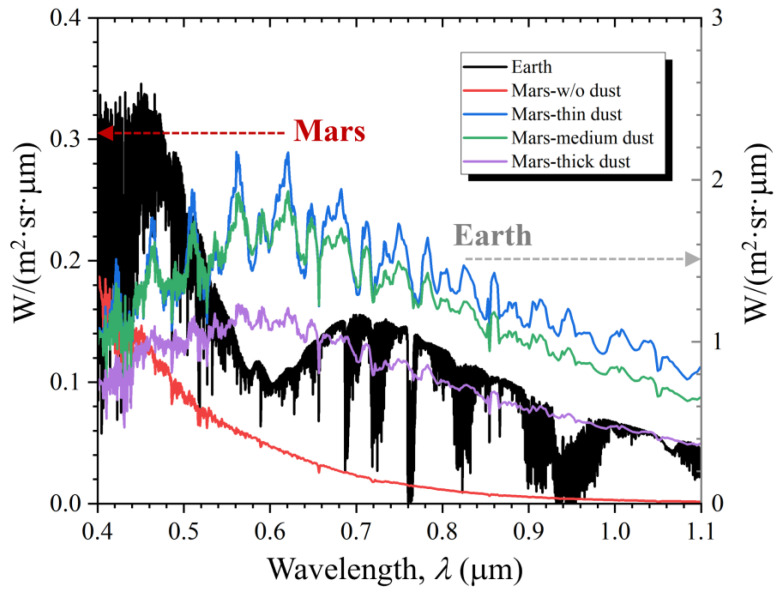
Dawn/dusk sky background radiance spectra on Mars under different dust conditions, compared with Earth (solar zenith angle = 90°, altitude = 0 km).

Figure 8 shows the spectral distribution of sky background radiance on Mars under different dust concentration conditions. For comparison with Earth, the figure also presents the corresponding results for Earth’s sky background radiance under similar conditions. It is important to note that Mars and Earth correspond to the left and right vertical axes in the figure, respectively. From the results in Figure 8, it can be observed that during dawn and dusk, the sky background radiance on Mars is roughly one order of magnitude smaller than on Earth. This is not only because Mars has a solar constant that is about one-third of Earth’s, but also because Mars’ dust particles obstruct the transmission of sunlight, reducing the proportion of sunlight that can be scattered and forming downwelling scattered light. This phenomenon is confirmed by the fact that the radiance at 400 nm, primarily caused by Rayleigh scattering, is higher than that under dusty conditions. Additionally, as the dust concentration increases, atmospheric transmittance decreases, and the corresponding radiance also decreases. Unlike the results presented in the previous section, a comparison of observations under thin and thick dust conditions reveals an entirely opposite relationship between atmospheric transmittance and sky radiance. In the previous analysis, thick dust conditions were selected to ensure that the optical parameters met the detection limits in the Martian dust environment. However, at this stage, both conditions should be analyzed separately.

**Figure 9:**
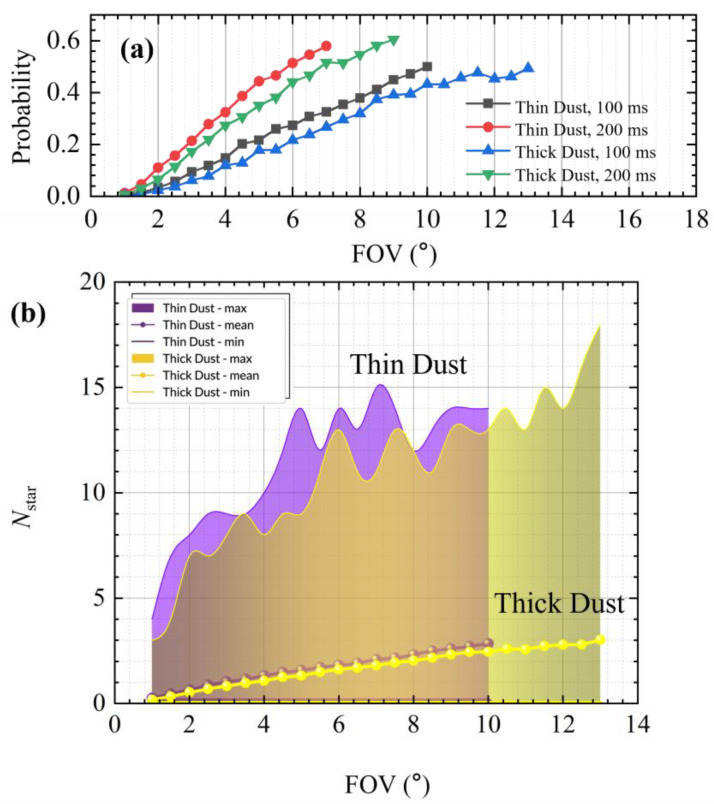
Dawn/dusk detection performance from 10,000 random observations on Mars.

To balance the single-star detection SNR with the requirement of detecting at least three stars within the FOV, the detection performance of the star sensor under dawn and dusk conditions was further investigated with respect to different FOV values. For the star sensor with an effective aperture of 22 mm, a simulation of 10,000 random detections across the entire sky was performed under dawn and dusk conditions. Figure 9a presents the detection success probability of having at least three stars with a SNR ≥ 6 within the FOV under different integration times, dust conditions, and FOVs. Figure 9b illustrates the maximum, minimum, and average number of stars with an SNR ≥ 6 under various dust conditions and FOV angles. Unlike nighttime detection, as the FOV angle increases, more sky background caused by dust scattering enters the lens, which can eventually lead to overexposure when the photoelectrons reach the camera’s full well capacity. Hence, the curves in Figure 9 terminate once the FOV angle reaches a certain threshold. From the results shown in the figure, increasing integration time does improve detection success rates, but it is accompanied by a smaller cutoff FOV angle. Higher dust concentration leads to lower sky background radiance, thus extending the cutoff FOV. To address this issue and enable a visible light star sensor to meet both nighttime detection needs and successfully conduct attitude determination during dawn and dusk conditions, there are methods that can be referenced.

For instance, the multi-frame accumulation technique involves taking a series of short exposures in quick succession [7,70]. However, since this technique is equivalent to increasing the total integration time, it is necessary to further evaluate star trailing and the dynamic performance of the star sensor [71,72,73,74]. Another method involves placing a filter in front of the lens to block light below a specific wavelength, thereby preventing overexposure and further improving the SNR, although this necessitates careful optimization of optical parameters [75]. Recent studies have also shown that the adoption of FOV gating technology can effectively enhance the SNR for all-time detection. Additionally, noise can be effectively suppressed from an image post-processing perspective [10,76], thereby enhancing the capability to detect stars with low SNRs.

## 4. Spatio-Temporal Assessment of Star-Sensor Detection Performance on the Martian Surface

### 4.1. Detection Performance Assessment at the Zhurong Rover Landing Site

**Figure 10:**
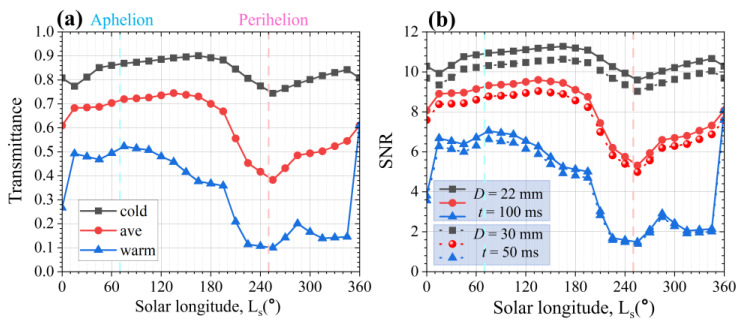
Seasonal variation in visible-band transmittance (**a**) and detection SNR (**b**) at the Zhurong landing site under different MCD v6.1 scenarios and optical configurations.

From a temporal perspective, the dust loading on Mars exhibits a quasi-periodic variation with Martian seasons, which are commonly characterized by the solar longitude *Ls*. The Martian dust season generally begins at *Ls* = 180° (autumnal equinox) and persists until *Ls* = 360° (vernal equinox). Figure 10a presents the variation in visible-band atmospheric transmittance of the dust layer with solar longitude at the landing site of the Zhurong rover. The three curves correspond to the cold scenario, climatology average solar scenario, and warm scenario cases provided by the MCD v6.1 model. The solar longitudes corresponding to Mars’aphelion (Ls = 71°) and perihelion (Ls = 251°) are indicated by blue and pink dashed lines, respectively. As shown in the figure, the visible-band transmittance of the Martian dust layer demonstrates a consistent seasonal periodicity, reaching relatively higher values near aphelion and lower values near perihelion. To further evaluate the performance of the previously designed optical parameters under realistic Martian surface observation conditions, Figure 10b illustrates the variation in detection SNR with solar longitude for the two optical configurations defined in Section 3.1, assuming a 6th-magnitude star with a color temperature of 8000 K. The results indicate that the *D* = 30 mm, *t* = 50 ms configuration fails to maintain SNR > 6 during the Martian dust season, with particularly pronounced degradation near perihelion. In contrast, for the sensor with an integration time of 100 ms, although a reduction in SNR still occurs during the Martian winter, especially near perihelion, the requirement SNR > 6 can be met for most periods throughout the Martian year. Therefore, when considering star sensor observations in the vicinity of the Zhurong rover landing site, the optical configuration of *D* = 22 mm and *t* = 100 ms is recommended, as it enables reliable detection under the majority of Martian dust conditions, provided that no regional or global dust storms occur.

The cold scenario, climatology average solar, and warm scenario cases from MCD v6.1 model used in Figure 10 are standard representations constructed based on dust conditions spanning Martian Years (MY) 24 to 31. In this study, Martian years are numbered following the convention proposed by Clancy et al. [77], where Martian Year 1 (MY 1) begins on 11 April 1955, defined by the northern spring equinox (Ls = 0°) [77]. These standard scenarios do not explicitly account for the occurrence of global dust storms on Mars. To further evaluate the detection performance of the star sensor at the Zhurong rover landing site and to validate the robustness of the proposed optical parameter design, it is necessary to assess the sensor performance using actual historical Martian dust data, including periods affected by global dust storms.

**Figure 11:**
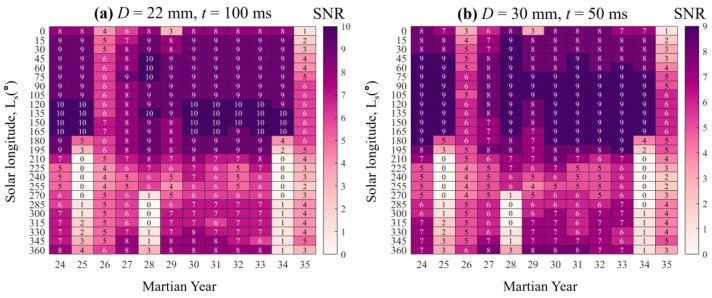
Seasonal SNR of an 8000 K 6th-magnitude star at the Zhurong landing site for MY 24–35 under two optical configurations.

Therefore, in this study, the detection SNR was calculated for a 6th-magnitude star with a color temperature of 8000 K based on the measured seasonal variations in dust optical depth for each Martian year from MY 24 to MY 35. Figure 11 presents the resulting star sensor SNR under different optical parameter configurations. The results clearly illustrate the variation in detection performance at the Zhurong rover landing site across two temporal dimensions: Martian year and Martian season.

As shown in Figure 11, significant degradation of SNR is observed during the autumn and winter seasons of MY 25, MY 28, and MY 34. Even when using the relatively optimal design parameters of *D* = 22 mm and *t* = 100 ms, the condition SNR > 6 cannot be satisfied throughout the entire Martian year, and in certain ranges of solar longitude, the SNR even drops to zero. In addition, MY 26 and MY 35 exhibit a year-round reduction in SNR. For the remaining Martian years, the SNR generally follows a consistent seasonal trend, with higher values in spring and summer and lower values in autumn and winter, while satisfying the detection requirement of SNR > 6 for most periods.

### 4.2. Global Detection Performance Evaluation on Mars

Due to the differences in elevation at various locations on the Martian surface, the distance that starlight travels from the Martian atmosphere to the entrance pupil of the star sensor will vary depending on the observation location. This variation in light path results in differences in optical transmittance at different locations on the Martian surface. In addition to the elevation differences, factors such as Martian climate and wind can also lead to variations in the dust and atmospheric conditions across the surface. When these factors are combined, they significantly influence the detection SNR, with the location of the star sensor on the Martian surface playing a crucial role.

**Figure 12:**
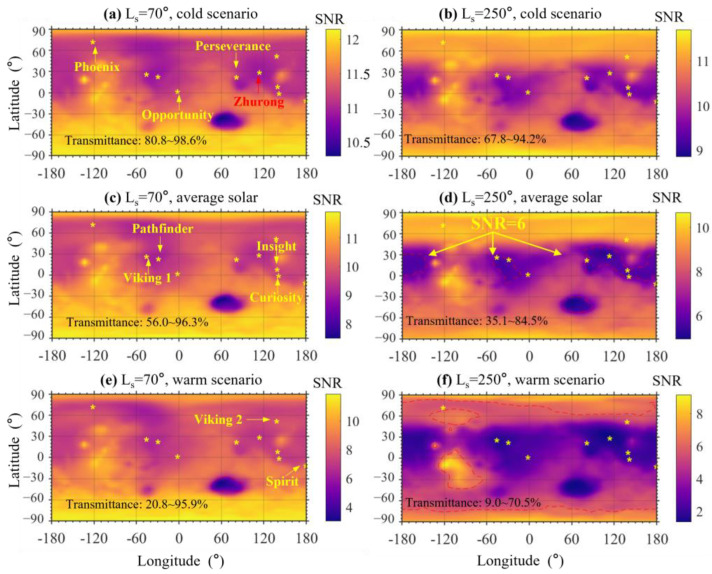
Global SNR maps for an 8000 K 6th-magnitude star at 0 km altitude (*D* = 22 mm, *t* = 100 ms) at aphelion and perihelion under three MCD v6.1 scenarios, with the SNR = 6 contour and 10 Mars landing sites marked.

Figure 12 marks the landing sites of 10 successful Mars missions—Zhurong, Perseverance, InSight, Curiosity, Phoenix, Opportunity, Spirit, Pathfinder, Viking 1, and Viking 2—using yellow stars. The figure also presents the detection SNR calculation for a 6th-magnitude star with a color temperature of 8000 K, based on a star sensor with an effective aperture of 22 mm and an integration time of 100 ms, located at 0 km altitude on the Martian surface. The results shown in the left and right columns correspond to conditions when Mars is at aphelion and perihelion, respectively. When Mars is at aphelion, both the cold scenario and average solar modes can achieve SNR > 6 across the entire globe, while in the warm scenario mode, the detection SNR decreases below 6 in the Elysium Planitia region. When Mars is at perihelion, the cold scenario mode still ensures global SNR > 6, while in the average solar mode, regions such as parts of the Amazonis Planitia, the southern part of the Arcadia Planitia, the Isidis Planitia, and the Elysium Chasm all experience areas with SNR < 6. Notably, the locations of the Pathfinder, Viking 1, Perseverance, InSight, and Curiosity rovers are in good agreement with the SNR = 6 contour lines. When Mars is at perihelion, the warm scenario mode shows that the detection SNR across most of the Martian surface drops below 6, with only the northern plateau, southern polar regions, the Valles Marineris, Olympus Mons, and the Tempe Terra region maintaining SNR > 6.

**Figure 13:**
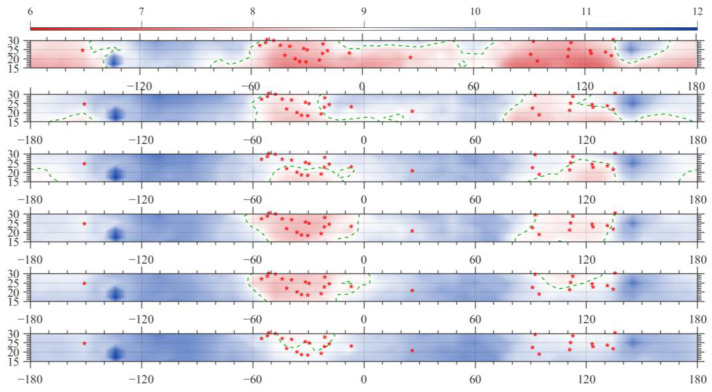
Warm-scenario global SNR distribution, with Tianwen-3 [25] candidate sites (red asterisks) and the SNR = 9 contour (green dashed line).

As illustrated in Figure 13, the detection performance near the ideal landing site of Tianwen-3 is significantly enhanced by the optimized design parameters. This configuration ensures a global SNR exceeding 6 in most scenarios. Nevertheless, under the warm scenario mode at Mars’ perihelion, some degradation is still observed, with the SNR falling below 6 in specific regions such as the Amazonis Planitia, Arcadia Planitia, and Isidis Planitia.

## 5. Earth Environment Detection Equivalence Analysis and Experimental Verification

Due to the strong path-integrated extinction and multiple scattering effects of suspended dust particles, a faithful experimental reproduction of the Martian dusty atmosphere would require an extended propagation path (on the order of kilometers) to capture the cumulative attenuation and scattered radiance characteristic of the actual Martian surface environment. Such a large-scale facility is impractical for routine laboratory implementation. A feasible and cost-effective approach is to construct a semi-physical simulation model that approximates the Martian atmospheric environment using a proportional scaling method. The following section presents the equivalence experimental scheme proposed for simulating Martian atmospheric and dust detection conditions in Earth environments, along with the rationale for conducting equivalence experiments using this method.

**Figure 14:**
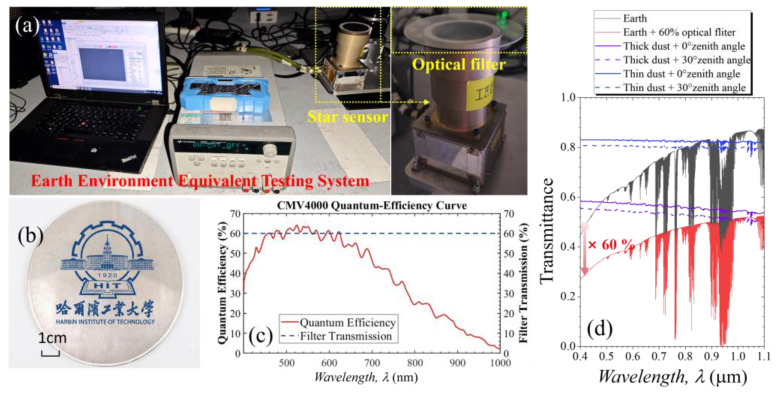
Earth-equivalence experiment: (**a**) system setup; (**b**) custom filter; (**c**) quantum efficiency and filter transmission spectra; (**d**) Earth–Mars atmospheric transmittance comparison under equivalent conditions.

Figure 14a shows a schematic diagram of the equivalence testing system in Earth’s environment. The system primarily consists of a star sensor, a host computer, a direct current power supply, and other components. The star sensor lens has an effective aperture of 22 mm, a FOV of 18°, and an optical system transmittance of 65%, using a CMV4000 visible light sensor. Notably, a customized filter (visible light transmittance of 60%) is positioned in front of the star sensor lens to attenuate starlight, which is equivalent to a 40% attenuation of Earth’s atmospheric transmittance. The filter employed in the experiment is a Neutral Density filter with a flat spectral response. The selection of 60% transmittance was a deliberate choice to establish a conservative ‘worst-case’ performance envelope for the detection system. As demonstrated in Figure 14d, the 60% attenuated Earth atmospheric transmittance is designed to be consistently lower than the transmittance on the Martian surface, even under the most severe ‘thick dust’ conditions across the visible spectrum. Ensuring the reliable performance of the star sensor under these artificially worsened Earth conditions provides a robust lower-bound verification that the proposed optical design possesses sufficient margin to handle the actual Martian dust environment. Figure 14b displays a physical image of the customized filter. Figure 14c shows CMV4000 Quantum-Efficiency Curve and filter transmission. Figure 14d presents the atmospheric transmittance of Earth, the transmittance after a 40% attenuation, as well as the atmospheric transmittance under Martian conditions for zenith angles of 0° and 30° in both thin and thick dust scenarios. From the results shown in the figure, it can be seen that in the visible light response band of the star sensor, the atmospheric transmittance under thin dust conditions is higher than that of Earth’s atmosphere, and under thick dust conditions, the atmospheric transmittance is higher than the 40% attenuated Earth’s atmospheric transmittance.

By comparing the target signal charge S at the output of the star sensor’s image sensor under different atmospheric transmittances on Mars and Earth, and using Equations (3)–(5), the following relationship is obtained:

(25) $$S_{earth}=\int_{\lambda_{1}}^{\lambda_{2}}\tau_{filter}(\lambda )\cdot \tau_{e}(\lambda )\cdot E_{m}(\lambda ,T)\cdot \pi \cdot {\left(\frac{D}{2}\right)}^{2}\cdot t\cdot \frac{1}{E_{\mathrm{ph}}}\cdot \eta (\lambda )d\lambda$$

(26) $$S_{mars}=\int_{\lambda_{1}}^{\lambda_{2}}\tau_{o}(\lambda )\cdot \tau_{m}(\lambda )\cdot E_{m}(\lambda ,T)\cdot \pi \cdot {\left(\frac{D}{2}\right)}^{2}\cdot t\cdot \frac{1}{E_{\mathrm{ph}}}\cdot \eta (\lambda )d\lambda$$

In the formula, $\tau_{filter}(\lambda )$ denotes the transmittance of the optical system (with a 60% filter); $\tau_{e}(\lambda )$ denotes Earth’s atmospheric transmittance; $\tau_{m}(\lambda )$ denotes Mars atmospheric transmittance (thin or thick dust conditions); η(λ) denotes the quantum efficiency.

This indicates that, under otherwise identical detection parameters, the target signal charge S at the output of the star sensor’s image sensor under Martian thin- and thick-dust conditions is higher than that in the Earth atmosphere and in the Earth atmosphere equipped with a 60% transmission filter. Therefore, the analysis results obtained for the Earth cases can be applied to the corresponding Martian dust conditions in an equivalent manner.

**Figure 15:**
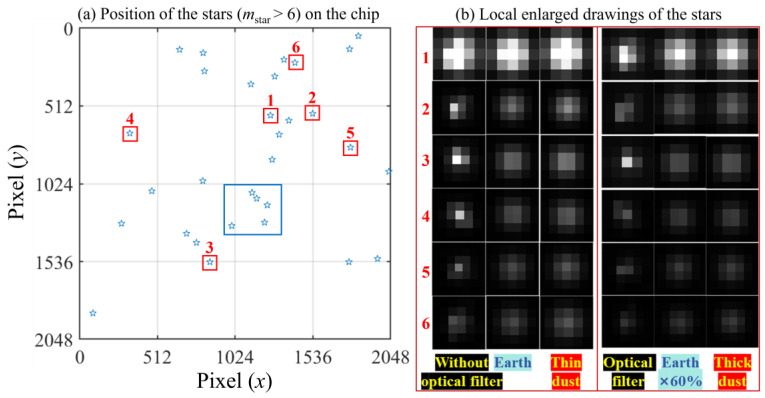
Comparison of raw and simulated star maps under Earth and Martian dust equivalence conditions.

Since the aforementioned optical parameter optimization design and performance evaluation methods are based on the same Mars atmospheric radiation simulation model and star sensor detection link simulation model, successfully comparing raw captured star maps with simulated star maps can confirm the accuracy of the simulation model and theory used in this paper. Figure 15a shows the positions of stars brighter than 6th magnitude in the FOV on the chip, with the optical axis pointing at coordinates (Latitude 31.4288°, Longitude 136.2494°, Roll Angle 105.0185°). Figure 15b presents a magnified local view of the six stars taken from (a) with a 100 ms integration time; these six stars were selected as representative of our observational results. From the results shown in the figure, it is clear that regardless of whether a filter is used, the gray levels in the raw captured star maps and simulated star maps in the Earth environment are quite similar. To further quantitatively analyze this, Table 1 provides the total gray sum (8-bit depth) of these 6 stars from raw captured star maps and simulated star maps in the Earth environment equivalence test, from a numerical perspective [78]. In the actual image acquisition, the defocus radius affects the size of the stellar spot; therefore, to ensure full coverage of the star spot pixels while staying consistent with the real captured images, the total gray sum for each star in Table 1 is calculated over a 5 × 5 pixel window centered at the brightest pixel of the star spot.

It can be seen that the error between the simulated results and raw captured results is generally within ±15%, with the maximum discrepancy being 13.9% as detailed in Table 1. The reasons for this error could be multifaceted: (1) Star Energy: The method used to calculate color temperature via BV numbers relies on a fitting formula, which inherently introduces some level of error. (2) Atmospheric Transmittance: The Earth atmospheric transmittance used in our simulation is based on a typical basic model. However, in real-world captures, the actual Earth atmospheric transmittance is influenced by various factors, leading to discrepancies with our chosen model. Atmospheric turbulence can also cause fluctuations in the energy of individual stars, causing some stars to appear brighter or dimmer. (3) Star Sensor Parameters: From Figure 15, it is clear that the star energy in raw captured star maps is more concentrated compared to simulated star maps. The selection of the speckle blur radius also influences this result. Star energy distribution typically follows an asymmetric normal distribution, and accurately simulating this asymmetry requires more detailed measurements. This is another reason why the error exists between our simulated star maps and raw captured star maps.

Although there is some level of error between the simulated results and the raw captured star maps due to various factors, from the perspective of optical parameter design and performance evaluation, this level of error is acceptable. It is sufficient to validate the correctness of the theory and methods on which this paper’s optical parameter design and performance evaluation are based.

Actually, from the comparison of atmospheric transmittance between Earth and Mars shown in Figure 14d, it can be inferred that under thin dust conditions on Mars, the star brightness should be slightly higher than in Earth’s environment, and under thick dust conditions on Mars, the star brightness should be slightly higher than in Earth’s environment with a 60% filter. The results in Figure 15b indeed demonstrate this pattern. To further clearly reveal this trend, Table 2 presents the SNR comparison for the six stars in both Earth-equivalent and Mars dust environments. The results indicate that under the same conditions, Mars’ detection SNR is about 10% higher in thin dust conditions compared to Earth, and about 20% higher in thick dust conditions compared to Earth’s environment with a 60% filter. Therefore, for well-optimized optical parameters in star sensors, successful detection in Earth’s atmospheric environment means the ability to achieve detection under thin dust conditions on Mars. Similarly, successful detection in Earth’s environment with a 60% filter implies the ability to detect successfully under thick dust conditions on Mars.

**Figure 16:**
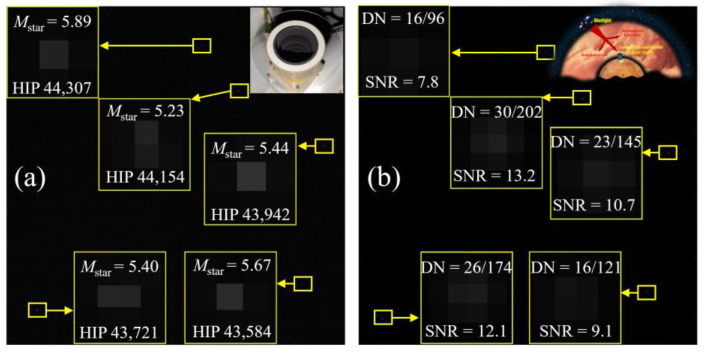
Raw captured star map with a filter and simulated star images under thick dust conditions on Mars.

Figure 16a presents a magnified local view of the star points within the yellow rectangular region outlined in Figure 15a, under the condition of a 60% filter. The figure provides the corresponding stellar magnitudes and star identifiers for the star points. From a visual grayscale observation, it can be difficult to determine whether these star points can be successfully detected. Figure 16b provides the grayscale (the brightness of the brightest pixel and the total grayscale of the star point) and the detection SNR for the stars under thick dust conditions. The results indicate that even for a magnitude 5.89 star, the brightness of the brightest pixel reaches 16 (8-bit depth), with an SNR of 7.8, confirming that it can be successfully detected.

The results presented in Figure 14, Figure 15 and Figure 16 and Table 1 and Table 2 demonstrate that the method of Earth environment equivalence analysis and experimental validation employed in this study is both reasonable and reliable. Repeated experiments have verified that successful detection can be achieved for any FOV orientation under Earth conditions with a 60% filter. This demonstrates that the selected optical design parameters are sufficient to support successful detection in Mars environments with thick dust conditions.

## 6. Conclusions

This study develops a spectral near-infrared atmospheric radiative transfer model for Martian dust environments based on the NCMCM and systematically characterizes the absorption, scattering, and multiple-scattering behavior produced by the combined effects of Martian dust and the rarefied atmosphere [79]. Using this model, the spectral atmospheric transmittance and the distribution of dawn and dusk sky background radiance are obtained as functions of wavelength and dust loading. The results show that Martian dust induces strongly wavelength-dependent attenuation of starlight in the near-infrared band, while the enhanced sky radiation under dawn and dusk conditions further degrades the imaging SNR, making it a key physical factor governing surface-based detection performance.

Building on these spectral radiative transfer results, the SNR characteristics of the imaging chain are evaluated, and the impacts of different optical parameters on surface-based detection performance are predicted for nighttime and dawn-dusk operating modes [80]. The analysis indicates that, despite the strong scattering and background radiation associated with Martian dust, appropriate configurations of aperture, FOV, and integration time can still provide sufficient starlight signals to ensure stable imaging. The results indicate that, with an effective aperture of 22 mm, an 18° FOV, and a 100 ms integration time, reliable nighttime detection can be achieved even under severe Martian dust conditions. Under thin dust conditions, an 8° FOV combined with a 22 mm aperture and a 200 ms integration time yields a dawn and dusk detection success rate exceeding 60%.

By using the MCD v6.1 model, the spectral optical transmittance of the Martian dust layer was evaluated, and the SNR at the Zhurong rover landing site was analyzed as a function of solar longitude. For a star sensor with an effective aperture of 22 mm and an integration time of 100 ms, the condition of SNR > 6 is still satisfied for most of the Martian year. The SNR at the ideal landing site of Tianwen-3 was also analyzed, showing that an SNR greater than 6 can be achieved under various Martian environmental conditions. Additionally, the radiance values observed by the Navcam were compared with the model’s simulated results, with an error of 7.83%. In addition, the proposed Earth–Mars radiative equivalence method is validated through grayscale comparisons among real Earth images, Earth-based simulations, and Mars-based simulations, demonstrating its effectiveness in both radiometric consistency and imaging conformity.

To further enhance the applicability of the model, future work will incorporate the effects of star refraction and wavefront perturbations induced by atmospheric turbulence [3], as well as the propagation of star sensor measurement uncertainties [81,82]. Moreover, considering that starlight transmission near the Martian surface is a typical imaging process through a scattering medium, inverse-imaging approaches based on machine learning are expected to further improve star image quality under dusty Martian conditions [83,84,85,86].

It is important to note that this study evaluates star sensor performance under simplified Martian dust conditions. The effects of water vapor, ice crystals, and ozone on radiation in the visible to near-infrared wavelength range are minimal and can therefore be neglected [41]. Future work may address these factors in further analyses. Additionally, this study focuses on a fixed observation platform on the Martian surface, where dynamic effects such as rover motion, jitter, and star trail formation are not considered to significantly impact the results.

## Figures and Tables

**Table 1:** Comparison of grayscale between real star images and simulated star images corresponding to six stars in Figure 15.

No.	Star ID	Magnitude	Earth Raw Image	Earth Simulation	(Simulation-Real)/ Real × 100	Earth Real Image (60% Filter)	Earth Simulation (Atmospheric Transmittance × 60%)	(Simulation-Real)/ Real × 100
1	45860	3.14	2555	2309	−9.6	1573	1439	−8.5
2	45688	3.82	761	779	2.4	442	466	5.4
3	43103	4.03	825	774	−6.2	490	464	−5.3
4	46146	4.47	603	563	−6.6	356	340	−4.5
5	44700	4.56	427	480	12.4	268	285	6.3
6	46952	4.54	410	467	13.9	270	282	4.4

**Table 2:** Comparison of SNR for six stars corresponding to Earth equivalent environment and Mars dust environment in Figure 15.

No.	Earth Simulation	Mars Simulation (Thin Dust)	(Mars-Earth)/ Earth × 100	Earth Simulation (Atmospheric Transmittance × 60%)	Mars Simulation (Thick Dust)	(Mars-Earth)/ Earth × 100
1	57	61	8	43	50	15
2	31	35	14	23	28	23
3	31	34	11	23	27	19
4	26	28	10	19	22	19
5	23	26	11	17	20	20
6	23	26	12	17	20	21

## Data Availability

The data are not publicly available due to institutional restrictions regarding data sensitivity.
